# Notch2 controls hepatocyte-derived cholangiocarcinoma formation in mice

**DOI:** 10.1038/s41388-018-0188-1

**Published:** 2018-03-16

**Authors:** Jingxiao Wang, Mingjie Dong, Zhong Xu, Xinhua Song, Shanshan Zhang, Yu Qiao, Li Che, John Gordan, Kaiwen Hu, Yan Liu, Diego F. Calvisi, Xin Chen

**Affiliations:** 1 Department of Bioengineering and Therapeutic Sciences, University of California, San Francisco, San Francisco, USA; 2 Dongfang Hospital, Beijing University of Chinese Medicine, Beijing, China; 3 307 Hospital of Academy of Military Medical Science, Beijing, China; 4 Department of Gastroenterology, Guizhou Provincial People’s Hospital, Guizhou, China; 5 Beijing Advanced Innovation Center for Food Nutrition and Human Health, College of Food Science and Nutritional Engineering, China Agricultural University, Beijing, China; 6 Department of Oncology, Beijing Hospital, National Center of Gerontology, Beijing, China; 7 Institut für Pathologie, Universitätsmedizin Greifswald, Greifswald, Germany

## Abstract

Liver cancer comprises a group of malignant tumors, among which hepatocellular carcinoma (HCC) and intrahepatic cholangiocarcinoma (ICC) are the most common. ICC is especially pernicious and associated with poor clinical outcome. Studies have shown that a subset of human ICCs may originate from mature hepatocytes. However, the mechanisms driving the trans-differentiation of hepatocytes into malignant cholangiocytes remain poorly defined. We adopted lineage tracing techniques and an established murine hepatocyte-derived ICC model by hydrodynamic injection of activated forms of AKT (myr-AKT) and Yap (YapS127A) proto-oncogenes. Wild-type, *Notch1*^*flox/flox*^, and *Notch2*^*flox/flox*^ mice were used to investigate the role of canonical Notch signaling and Notch receptors in AKT/Yap-driven ICC formation. Human ICC and HCC cell lines were transfected with siRNA against Notch2 to determine whether Notch2 regulates biliary marker expression in liver tumor cells. We found that AKT/Yap-induced ICC formation is hepatocyte derived and this process is strictly dependent on the canonical Notch signaling pathway in vivo. Deletion of *Notch2* in AKT/Yap-induced tumors switched the phenotype from ICC to hepatocellular adenoma-like lesions, while inactivation of *Notch1* in hepatocytes did not result in significant histomorphological changes. Finally, in vitro studies revealed that Notch2 silencing in ICC and HCC cell lines down-regulates the expression of Sox9 and EpCAM biliary markers. Notch2 is the major determinant of hepatocyte-derived ICC formation in mice.

## Introduction

Primary liver cancer is the second most common cause of cancer mortality in the world, with increasing incidence globally [1, 2]. Hepatocellular carcinoma (HCC) and intrahepatic cholangiocarcinoma (ICC) are the two most prevalent liver tumor types. Most ICCs are diagnosed at advanced stage and only a few patients are suitable for surgery at the time of diagnosis. For patients with inoperable ICC, very limited treatment options exist. According to the American Cancer Society (www.cancer.org), the 5-year survival rate for ICC patients with localized disease is ~15%, and only 2% for patients with distal metastasis. ICC has been traditionally considered to be derived from biliary epithelial cells (BEC). However, recent studies have indicated that adult hepatocytes can transdifferentiate into BEC-like cell in various chronic liver diseases, which then may further develop into malignant cells [3, 4]. In accordance with this hypothesis, recent epidemiology studies have shown that in Western countries, where biliary tract infection rate is extraordinarily low, chronic infections by hepatitis B or C virus as well as alcohol abuse are major risk factors for ICC [5], as described for HCC. Studies from our and other laboratories also confirmed that ICC can originate from mature hepatocytes in mice following activation of the Notch signaling [6, 7].

Notch is a highly conserved pathway during development. This pathway is critical for biliary cell coordination and tubule formation [8]. The structure, homeostasis, and carcinogenesis of the liver relies on the Notch cascade [9, 10]. In mammals, canonical Notch pathway consists of four receptors (Notch1, Notch2, Notch3, and Notch4) and mainly two types of ligands, Serrate/Jagged (Jagged1 and Jagged2) and Delta-like (DLL1, DLL3, and DLL4) [11–13]. This cascade is activated by direct cell–cell interaction, with subsequent cleavage of the Notch receptor extracellular domain (NECD). This structural change leads to the release of the Notch intracellular domain (NICD), which translocates into the nucleus and recruits coactivators, such as Mastermind-like proteins (MAML1, MAML2, or MAML3). Together with the recombinant signal-binding protein for immunoglobulin kappa J region (RBPJ) transcription factor, they form the transcription complex responsible for the induction of Notch target genes [9, 14]. The most studied Notch signaling targets are hairy/enhancer of split (Hes) and hairy/enhancer of split related with YRPW motif (Hey) families. Another emerging target is Notch-regulated ankyrin repeat protein (Nrarp), which is activated by the RBPJ-dependent Notch pathway [15].

Both Notch1 and Notch2 receptors are expressed in the liver, but whether they play distinct or redundant roles along hepatocarcinogenesis remains an unanswered issue. Notch1 is considered a tumor suppressor in HCC [16, 17], but a bona fide oncogene in ICC [18]. Deprivation of *Notch1* results in continuous proliferation of hepatocytes [19]. Notch2, on the other hand, seems to be crucial for the differentiation of BECs and is required for normal perinatal and postnatal intrahepatic bile duct (IHBD) development [20, 21]. The canonical Notch2 signaling can determine biliary cell fate of not only embryonic hepatoblasts, but also mature hepatocytes [22]. Furthermore, the expression of Notch2 is more often observed in well-differentiated ICC, indicating its role in biliary tumor cell differentiation [23]. In a recent study [24], we found that anti-Notch2 treatment can reduce both HCC and ICC tumor load induced by AKT and neuroblastoma RAS viral oncogene homolog (NRas) oncoproteins, whereas Notch1 suppression decrease HCC and augments ICC occurrence. This body of evidence suggests the conflicting functions of Notch1 and Notch2 receptors in different contexts.

Formerly, most of the studies regarding Notch1 and Notch2 receptors aimed at their overexpression rather than their suppression. In the present study, we sought to better define the cell functions of Notch1 and Notch2 receptors in mice by loss-of-function experiments. Thus, we generated a mouse model characterized by biliary trans-differentiation of hepatocytes, and investigated whether this cellular event depends on autocrine Notch1 or Notch2 signaling. Specifically, we overexpressed activated forms of v-akt murine thymoma viral oncogene homolog (myr-AKT) and yes-associated protein (YapS127A) genes in hepatocytes by hydrodynamic injection (AKT/Yap) [25]. By adopting lineage tracing technology [6], we show that AKT/Yap-induced ICC formation is hepatocyte derived and this process depends on the canonical Notch signaling pathway. Furthermore, we found that Notch2 is the major driver of the biliary phenotype in AKT/Yap tumors, whereas inactivation of Notch1 slightly delays tumor development without affecting the histological features of AKT/Yap ICC lesions.

## Results

### AKT/Yap-induced ICCs originate from hepatocytes

**Fig. 1:**
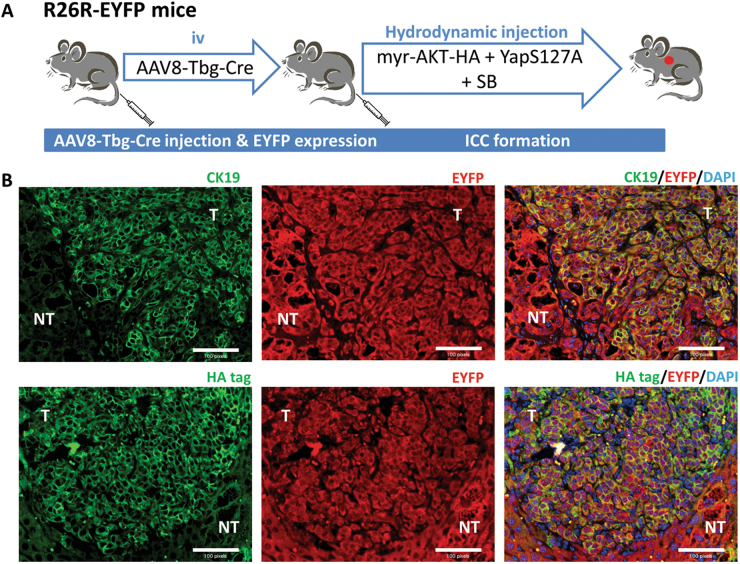
AKT/Yap-induced intrahepatic cholangiocarcinomas (ICC) derived from hepatocytes. **a** Study design. AAV-Tbg-Cre was injected intravenously to generate EYFP expression. AKT and Yap were co-injected hydrodynamically to induce tumor development. **b** The upper panels show immunofluorescence of CK19 and EYFP. The lower panels are representative staining of HA tag and EYFP. Note that EYFP was only expressed in hepatocytes, while surrounding mesenchymal cells did not show red fluorescence. Tumor is labeled as T and non-tumor is labeled as NT. Magnification: 100×

To determine whether ICC cells induced by AKT/Yap co-expression originate from mature hepatocytes, we applied the hepatocyte fate tracing model as previously described [6, 26]. Specifically, we injected adeno-associated virus encoding Cre-recombinase under the control of hepatocyte-specific thyroxine-binding globulin (Tbg) promoter (AAV8-Tbg-Cre) into mice that carry EYFP disrupted by a floxed stop codon in the ubiquitously expressed Rosa26 locus (*R26R-EYFP* mice). This procedure leads to the activation of hepatocyte-specific EYFP expression after 1 week of AAV injection. We applied hydrodynamic tail vein injection of hemagglutinin (HA) tagged AKT and YapS127 plasmid (AKT/Yap), along with Sleeping Beauty (SB) plasmids to initiate ICC development (Fig. 1a). Immunofluorescence (IF) staining showed that all tumor cells were positive for the BEC marker CK19, hepatocyte lineage marker EYFP, as well as ectopically expressed HA tagged AKT (Fig. 1b). The results demonstrate that AKT/Yap co-expression induces hepatocyte-derived ICCs in the mouse liver.

### Notch signaling cascade is activated in AKT/Yap ICC

**Fig. 2:**
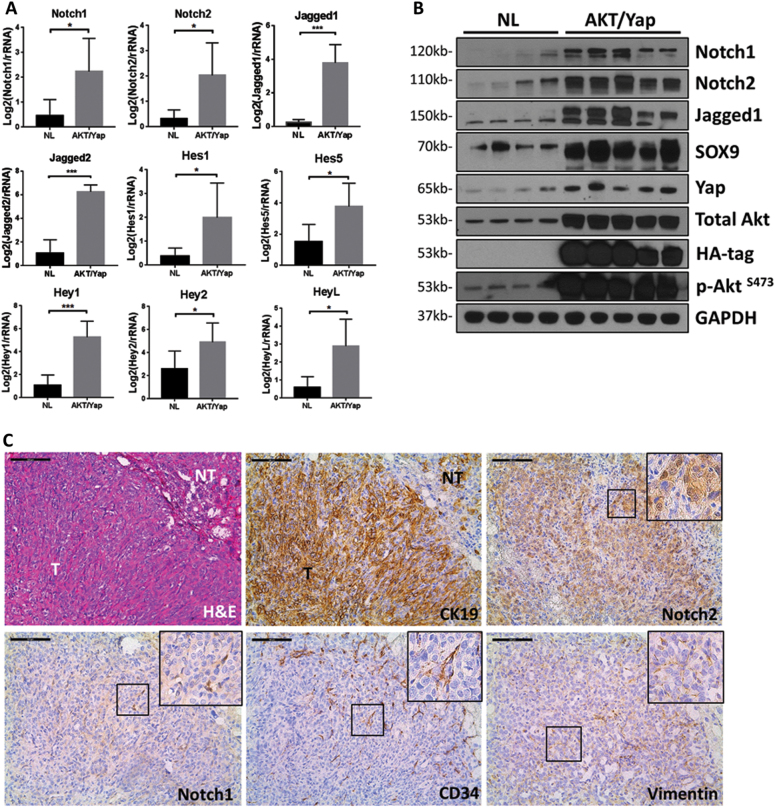
Notch signaling activation in intrahepatic cholangiocarcinoma (ICC) from AKT/Yap mice. **a** Notch signaling activation at transcriptional level is presented by qPCR. Data are analyzed and normalized using the −ΔΔCt method and presented as mean ± SD; NL represents normal liver; *n* = 6, *p < 0.05; **p < 0.01; ***p < 0.001. **b** Western blot analysis of relative protein expression in normal liver and tumor samples. GAPDH was used as a loading control. **c** Hematoxylin and eosin (H&E) staining and immunohistochemistry (IHC) of AKT/Yap-induced tumors. CK19 and Ki67 showed the ICC nature and the proliferative features of these tumors, respectively. Notch2 immunolabeling was localized in the cytoplasm and nucleus of ICC cells. Notch1 positive cells exhibited the same staining pattern of CD34 positive cells, which are mostly endothelial cells. Magnification: 200×; Scale bar: 100 µm

As the canonical Notch signaling has been implicated in cholangiocarcinogenesis [14, 27], especially hepatocyte-derived ICC formation [6, 7], we evaluated whether the Notch cascade is activated in AKT/Yap ICC tumors. We found that AKT/Yap ICCs express higher mRNA levels of Notch receptors (Notch1 and Notch2), Notch ligands (Jagged1 and Jagged2), and canonical Notch target genes (Hes1, Hes5, Hey1, Hey2, and HeyL) when compared to normal liver (Fig. 2a). Western blot analysis confirmed the upregulation of Notch1, Notch2, Jag1 as well as the BEC marker Sox9 in AKT/Yap tumors (Fig. 2b). To further characterize the specific cell type(s) within the tumors that express Notch receptors, Notch1 and Notch2 immunohistochemistry (IHC) was performed. We observed Notch2 immunoreactivity in the cytoplasm and nucleus of tumor cells, with no Notch2 expression being detected in mesenchymal cells. In contrast, Notch1 immunolabeling could be easily observed in cells surrounding the tumor nodules, similar to that of CD34, which marks endothelial cells (ECs) (Fig. 2c). Upon longer DAB (3,3′-diaminobenzidine) incubation time, we could detect weak cytoplasmic and/or membranous staining of Notch1 in tumor cells, as well as in surrounding non-neoplastic hepatocytes (Sup Fig. 1).

Altogether, our data underline the activation of the Notch cascade in AKT/Yap ICC. Specifically, Notch2 is strongly expressed in ICC cells, whereas Notch1 is predominantly expressed in the cells of the tumor microenvironment.

### AKT/Yap-induced ICC depends on the canonical Notch pathway

**Fig. 3:**
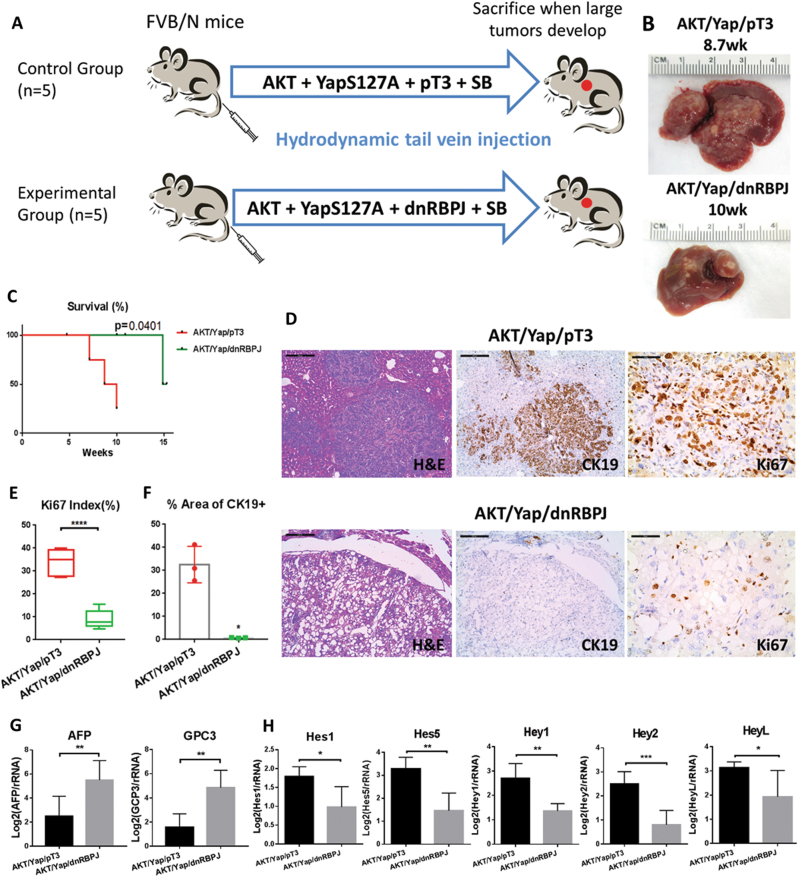
Intrahepatic cholangiocarcinoma (ICC) formation depends on canonical Notch pathway in AKT/Yap mice. **a** Study design. **b** Gross image of livers from each group. **c** Survival analysis of mice bearing AKT/Yap/pT3 (*n* = 5) and AKT/Yap/dnRBPJ (*n* = 5) tumors as assessed by the Kaplan–Meier survival method. **d** Upper panels show H&E and immunohistochemistry of AKT/Yap/pT3 mice, and lower panels that of AKT/Yap/dnRBPJ mice, respectively. Scale bar: 100 μm for 200×; 50 μm for 400×. **e** The percentage of Ki67 positive cells in the two groups (*n* = 5 per each cohort) was analyzed using Image Pro Plus. **f** Percentage of CK19 positive area in the two groups is displayed. Area fraction was analyzed with the ImageJ software; *n* = 3 per each group. **g** Relative mRNA expression of AFP and GPC3 was analyzed and normalized using the −ΔΔCt method. **h** Relative expression of Notch target genes is shown. All data are presented as mean ± SD. *p < 0.05; **p < 0.01; ***p < 0.001; **** *p* < 0.0001

Next, we tested whether the canonical Notch pathway is a major pathogenic player in AKT/Yap-driven cholangiocarcinogenesis. For this purpose, we co-expressed AKT and Yap plasmids with a dominant negative form of recombination signal-binding protein for immunoglobulin kappa J (dnRBPJ) in FVB/N mice, which will be referred to as AKT/Yap/dnRBPJ (Fig. 3a). RBPJ is a downstream transcription factor that constitutes the DNA-binding site of the Notch transcription complex. Expression of dnRBPJ has been shown to effectively block the canonical Notch signaling [28, 29]. Additional mice were injected with AKT and Yap with pT3-EF1α empty vector as control (AKT/Yap/pT3) (Fig. 3a). Of note, we found that blocking the canonical Notch cascade significantly delayed AKT/Yap-induced liver tumor formation and prolonged mouse survival (Sup Fig. 2 and Fig. 3b,c). Consistently, Ki67 index significantly decreased in AKT/Yap/dnRBPJ liver tumors when compared to that in AKT/Yap/pT3 liver tumors (Fig. 3d,e), implying that tumor cell proliferation was down-regulated by blocking the canonical Notch signaling. Most importantly, histological analysis revealed that while AKT/Yap/pT3 tumors consisted of pure ICC, as also indicated by strong immunoreactivity for the CK19 biliary marker (Fig. 3d,f), AKT/Yap/dnRBPJ liver tumors consisted mostly of hepatocellular adenomas and few HCC lesions. These observations were further underscored by increased levels of tumor hepatocellular markers, such as alpha-fetoprotein (AFP) and glypican 3 (GPC3), in AKT/Yap/dnRBPJ tumors (Fig. 3g). As expected, the downstream targets of the Notch signaling were down-regulated in AKT/Yap/dnRBPJ mice (Fig. 3h), thus substantiating the effective blockade of this cascade by dnRBPJ overexpression.

Taken together, our study demonstrates that the canonical Notch signaling is required for AKT/Yap-induced hepatocyte-derived ICC formation in mice.

### Limited effects of cell autonomous Notch1 signaling in AKT/Yap-induced ICC formation in mice

**Fig. 4:**
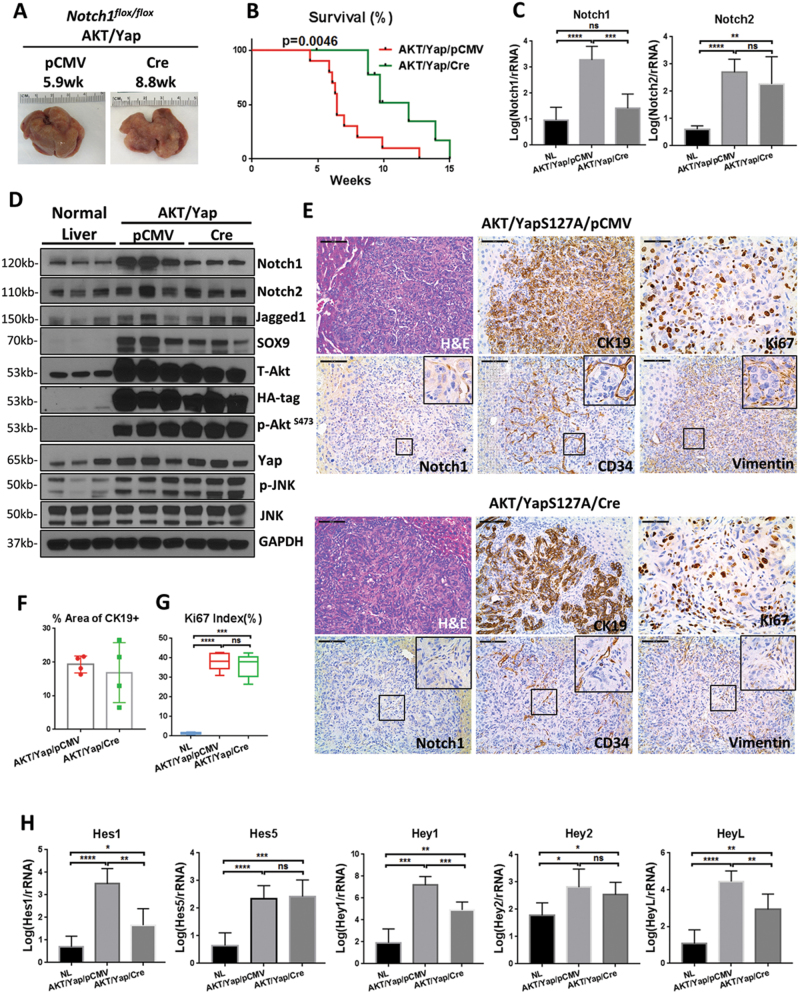
Notch1 inactivation does not prevent ICC formation in AKT/Yap mice. **a** Gross images of livers from AKT/Yap/pCMV and AKT/Yap/Cre mice. **b** Survival analysis of *Notch1*^*flox/flox*^ mice bearing AKT/Yap/pCMV (*n* = 10) and AKT/Yap/Cre (*n* = 10) tumors using the Kaplan–Meier survival method. **c** Relative mRNA expression of Notch1 and Notch2 was analyzed and normalized using the −ΔΔCt method and presented as mean ± SD; NL represents normal liver; *n* = 6. **d** Western blot analysis of normal liver, AKT/Yap/pCMV, and AKT/Yap/Cre mice. GAPDH was used as a loading control. **e** H&E and immunohistochemical staining of AKT/Yap/pCMV (upper panels) and AKT/Yap/Cre (lower panels) mice. Several sections were enlarged for a better view on the expression of Notch1, CD34, and Vimentin. Scale bar: 100 μm for 200×; 50 μm for 400×. **f** Quantification of CK19+ area percentage is displayed, *n* = 4. **g** Percentage of Ki67 positive nuclei was analyzed in normal liver (*n* = 5), AKT/Yap/pCMV (*n* = 5), and AKT/Yap/Cre (*n* = 5) mice. **h** Relative expression of Notch target genes is shown; *n* = 6. All data are presented as mean ± SD. ns *p* > 0.05; *p < 0.05; **p < 0.01; ***p < 0.001; ****p < 0.0001

Next, we investigated whether Notch1 or Notch2 was the major Notch receptor mediating AKT/Yap-induced ICC development. As our study shows the low expression of Notch1 in normal hepatocytes and ICC tumor cells (Sup Fig. 1), we first investigated the cell autonomous role of Notch1. Thus, *Notch1*^*flox/flox*^ mice were hydrodynamically injected with AKT, YapS127A, and pCMV/Cre plasmids to allow the expression of AKT/Yap oncogenes in *Notch1* knockout hepatocytes (AKT/Yap/Cre; Sup Fig. 3). *Notch1*^*flox/flox*^ mice injected with AKT, Yap, and pCMV (empty vector) were used as control (AKT/Yap/pCMV; Sup Fig. 3). Hepatocyte-specific ablation of *Notch1* delayed AKT/Yap-induced liver tumor development (Fig. 4a,b). qRT-PCR and Western blot analyses demonstrate that Notch1 mRNA and protein expression were both lower in AKT/Yap/Cre mouse liver tumors than that in control AKT/Yap/pCMV tumors (Fig. 4c,d). However, substantial Notch1 expression could still be observed in AKT/Yap/Cre tumors, suggesting that most of Notch1 was not expressed in tumor cells, but in other cell types within the ICC lesions. Consistent with this observation, IHC revealed that Notch1 could still be detected in AKT/Yap/Cre mouse liver tumors, and most likely in CD34(+) ECs (Fig. 4e). Histological analysis showed that tumor-specific ablation of *Notch1* did not change tumor morphology: indeed, ICC lesions were found throughout the liver and no hepatocellular tumors, including hepatocellular adenoma and HCC, were observed in both AKT/Yap/Cre and AKT/Yap/pCMV *Notch1*^*flox/flox*^ mice (Fig. 4e). The overall ICC tumor burden, as measured by the percentage of CK19(+) liver area, did not differ in the two cohorts (Fig. 4f). Similarly, no difference in cell proliferation was observed (Fig. 4g). Finally, loss of *Notch1* in tumor cells did not affect the protein expression of Notch2, Jag1, and BEC marker Sox9 as well as mRNA levels of Notch targets, including Hes5 and Hey2 (Fig. 4c,d,h). Loss of Notch1 instead decreased the expression of Hes1, Hey, and HeyL (Fig. 4h).

In summary, our study demonstrates that Notch1 expression is low in AKT/Yap tumor cells, and ablation of *Notch1* in hepatocytes delays ICC tumorigenesis. However, the cell autonomous Notch1 signaling is neither a major determinant of activated canonical Notch cascade nor the key driver for AKT/Yap-induced ICC development in mice.

### Cell autonomous Notch2 deprivation is required for AKT/Yap-induced ICC development in mice

**Fig. 5:**
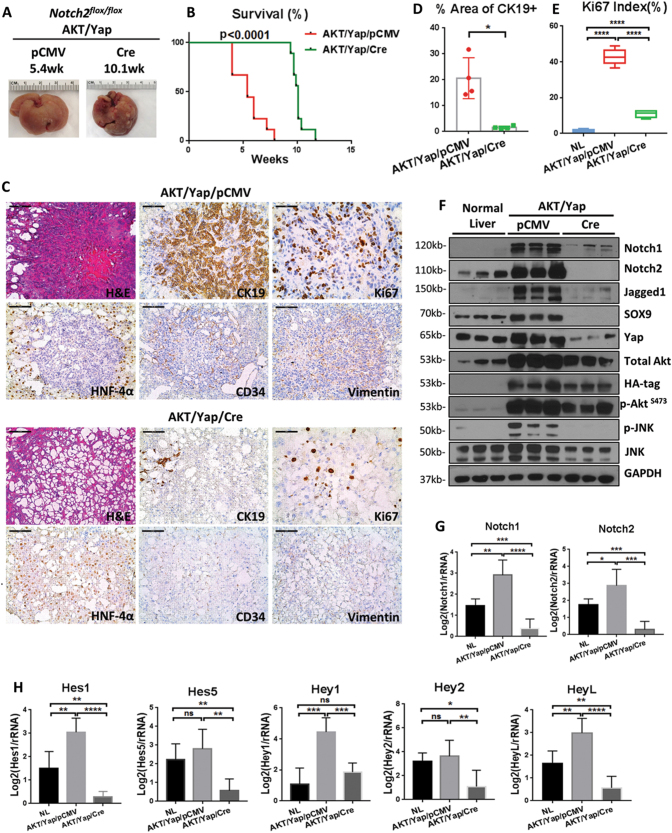
Notch2 inactivation suppresses ICC development in AKT/Yap mice. **a** Gross images of AKT/Yap/pCMV and AKT/Yap/Cre livers. **b** Survival analysis of *Notch2*^*flox/flox*^ mice bearing AKT/Yap/pCMV (*n* = 9) and AKT/Yap/Cre (*n* = 9) tumors. **c** H&E and immunohistochemistry of AKT/Yap/pCMV (upper panels) and AKT/Yap/Cre (lower panels) mice. Several sections were enlarged for optimal vision. Scale bar: 100 μm for 200×; 50 μm for 400×. **d** CK19 staining was quantified and represents the percentage of positive staining area of the whole area in the two groups, *n* = 4. **e** Percentage of Ki67 positive cells was analyzed in normal liver (*n* = 5), AKT/Yap/pCMV (*n* = 6), and AKT/Yap/Cre (*n* = 6) livers. **f** Western blotting of normal liver, AKT/Yap/pCMV, and AKT/Yap/Cre livers in *Notch2*^*flox/flox*^ mice. GAPDH was used as a loading control. **g** Relative expression of Notch1 and Notch2 using qPCR. Data were analyzed and normalized using the −ΔΔCt method, *n* = 6. **h** Relative expression of Notch signaling target genes using qPCR; *n* = 6. All data are presented as mean ± SD. ns *p* > 0.05; *p < 0.05; **p < 0.01; ***p < 0.001; ****p < 0.0001

In light of the pivotal role of Notch2 signaling in biliary cell fate determination [22], we investigated the function of Notch2 in AKT/Yap ICC lesions using conditional *Notch2*^*flox/flox*^ mice (Sup Fig. 4). Specifically, we hydrodynamically injected AKT, Yap, and Cre into *Notch2*^*flox/flox*^ mice (AKT/Yap/Cre). *Notch2*^*flox/flox*^ mice injected with AKT, Yap, and pCMV empty vector (AKT/Yap/pCMV) were used as control. Ablation of *Notch2* significantly delayed AKT/Yap-induced ICC development in mice, although eventually all mice developed lethal burden of liver tumors and were required to be euthanized (Fig. 5a,b). Histologically, ICC lesions could be found throughout the liver parenchyma of AKT/Yap/pCMV mice, while no hepatocellular lesions were identified. Strikingly, ablation of *Notch2* resulted in mostly hepatocellular adenomas with some HCC lesions in the liver, while no ICC lesions could be detected (Fig. 5c). These findings were further validated via IHC of BEC marker CK19 and hepatocyte-specific marker HNF-4α (Fig. 5c,d). Proliferation of tumor cells was significantly reduced following *Notch2* depletion, as indicated by Ki67 index (Fig. 5e). Western blot analysis confirmed the loss of Notch2 in the liver tumor lesions (Fig. 5f). In addition, mRNA levels of the canonical Notch targets, including Hes1, Hes5, Hey1, Hey2, and HeyL were remarkably dowregulated following *Notch2* deletion, as assessed by qRT-PCR analysis (Fig. 5g,h). These data indicate that Notch2 is the major regulator of the canonical Notch cascade in this mouse model. Interestingly, Notch1 expression was also decreased upon *Notch2* ablation (Fig. 5f,g). Immunolabeling of CD34 and Vimentin in the tumor sections revealed that ECs and fibroblast cells were less numerous in AKT/Yap/Cre lesions (Fig. 5c). Since Notch1 is most likely expressed dominantly in ECs, the reduced ECs may account for the decreased Notch1 expression in AKT/Yap/Cre lesions.

Recent experimental data point to the JNK signaling as a critical modulator of biliary differentiation and proliferation [30]. Accordingly, we found that the JNK pathway was effectively inactivated following Notch2 (Fig. 5f) but not Notch1 (Fig. 4d) deletion, thus suggesting that the JNK cascade could be a crucial downstream effector of Notch2 in ICC.

Altogether, these data indicate that Notch2 is essential for Akt/Yap-induced ICC development.

### Deletion of Notch2 undermines the biliary properties in vitro

**Fig. 6:**
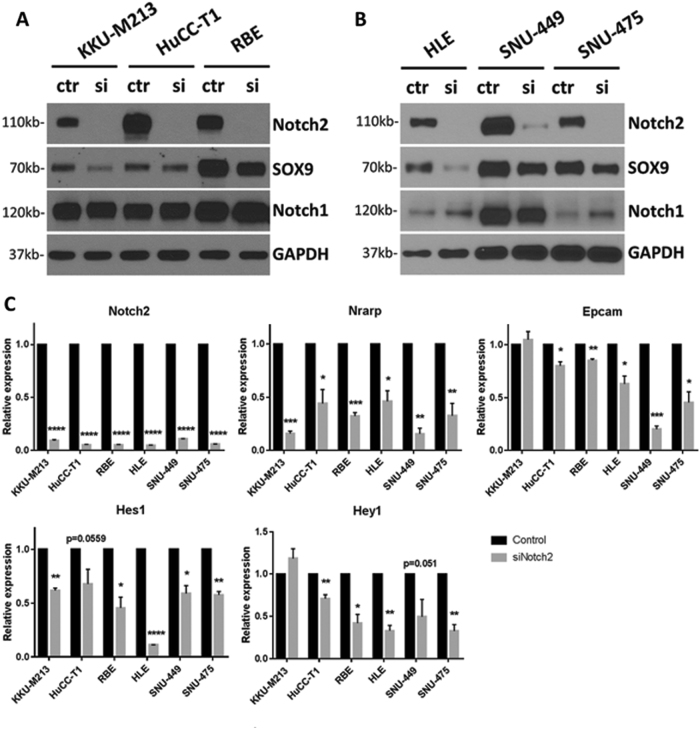
Inactivation of Notch2 in human intrahepatic cholangiocarcinoma (ICC) and hepatocellular carcinoma (HCC) cell lines. **a** and **b** Western blot analysis shows the complete inhibition of Notch2 expression in ICC and HCC cell lines transfected with siNotch2. Control is presented as ctr and siNotch2 group is indicated as si. GAPDH was used as a loading control; *n* = 3. **c** qPCR analysis of Notch2, Nrarp, Epcam, Hes1, and Hey1. Data are analyzed and normalized using the −ΔΔCt method, *n* = 3. All data are presented as mean ± SD. ns *p* > 0.05; *p < 0.05; **p < 0.01; ***p < 0.001; ****p < 0.0001

The studies in mice suggest the possibility that Notch2 controls BEC-like cell fate in human liver tumor cells. Thus, we tested whether silencing of Notch2 affected BEC marker expression in human HCC and ICC cell lines. A total of three ICC cell lines (KKU-M213, HuCC-T1, and RBE) and three HCC cell lines (HLE, SNU-449, and SNU475) were transfected with scrambled control siRNA or siNotch2. As expected, Notch2 expression was impaired following siNotch2 treatment in both ICC and HCC cell lines (Fig. 6a,b). Also, knockdown of Notch2 was paralleled by decreased SOX9 expression as well as by decline of canonical Notch targets, including Hes1, Hey1, and Nrarp (Fig. 6c). Transcriptional levels of Epcam were significantly reduced in all HCC cell lines and two ICC cell lines.

In summary, our study indicates that Notch2 expression, at least partially, controls BEC fate gene expression in human HCC and ICC tumor cells.

## Discussion

ICC is a malignant form of liver tumor lacking effective treatment options. The cellular origin of ICC is currently a matter of debate. Previously, it was believed that ICC arises from BEC of the liver. This is most likely the case in patients where a predisposing risk factor is limited to the biliary ducts, such as infections by *Clonorchis sinensis*, choledocholithiasis, or choledochal cysts. However, in Western and East Asian countries, where inflammatory processes affecting the biliary tract are uncommon, HBV and HCV infection and alcohol abuse are the main risk factors for ICC, as for HCC [5]. Since HBV and HCV viruses do not infect BECs, how ICC develops in the context of chronic hepatocyte injury remains a matter of debate. Presumably, hepatocytes are the cells of origin of this subset of human ICCs. Previous studies have shown the extraordinary plasticity of mature hepatocytes, which can transdifferentiate into BECs upon injury [4, 31, 32]. In the presence of additional oncogenic and/or mutagenic stimuli, it is possible that these trans-differentiated BECs further transform into ICC cells. Besides hepatocytes, several other cell types can also undergo trans-differentiation, leading to cancer formation [33]. One of such examples is pancreatic ductal adenocarcinoma (PDAC) [34, 35]. Like ICC, PDAC was thought to originate from ductal cells within the pancreas. However, recent studies have challenged this assumption, showing that acinar cells (the major parenchymal cells in the pancreas) are able to transdifferentiate into ductal cells. This process, also known as acinar-to-ductal metaplasia, can facilitate pancreas regeneration after injury [34, 35]. These metaplastic cells have been found to be precursors of pre-neoplastic PanIN lesions, which can further progress to PDAC [34, 35]. In this study, we show that AKT/Yap-induced ICCs arise from mouse hepatocytes. Our previous investigation has demonstrated the frequent concomitant activation of AKT and Yap signaling cascades in human ICCs [25]. Thus, we believe that the AKT/Yap mouse model is highly relevant to study human cholangiocarcinogenesis, especially in the context of chronic liver injury where hepatocytes may in fact be the target of transformation.

The molecular mechanisms underlying the development of hepatocyte-derived ICC remain poorly understood. Previous studies from our and other groups identified the canonical Notch cascade as a critical driver in hepatocyte-derived ICC [6, 7]. However, all these studies were based on gain of function of Notch receptors, aiming to demonstrate that activation of Notch is sufficient to promote ICC formation. Whether Notch is required for hepatocyte-driven ICC development, and if so, which Notch receptor is responsible for this process have not been previously investigated. In this study, by co-expressing AKT and Yap oncogenes with dnRBPJ, which blocks the canonical Notch cascade [28, 29], we convincingly proved that the endogenous Notch signaling is required for hepatocyte-derived ICC. It is worth to note that Notch was found to be required for acinar-to-ductal metaplasia in a KRas-driven PDAC murine model [36]. In addition, a recent study showed that activation of endogenous Notch leads to trans-differentiation of neuroendocrine to non-neuroendocrine cells in small-cell lung cancer cells [37]. Together these studies point to Notch signaling as an important cell-fate determinant in multiple tumor types.

Furthermore, we investigated which Notch receptor is responsible for Notch-dependent-ICC development, focusing on Notch1 and Notch2 receptors. Using conditional *Notch1* and *Notch2* KO mice, we demonstrated that loss of Notch1 only moderately delays AKT/Yap ICC development. However, ICC lesions eventually occurred in all mouse livers depleted of *Notch1*. In striking contrast, when *Notch2* was knocked-out in hepatocytes, not only it led to delayed tumor development, but also resulted in the exclusive formation of hepatocellular adenoma and HCC-like lesions, thus recapitulating the phenotype observed following the inhibition of the canonical Notch signaling. These observations indicate that the cell autonomous Notch2 is required for hepatocyte–BEC trans-differentiation and ICC formation in mice. In addition, using human HCC and ICC cell lines, we showed that silencing of Notch2 decreased the expression of the BEC marker Sox9. We then analyzed Notch2 expression in relationship to Sox9 in a human TCGA dataset containing 59 non-tumor livers and 36 human ICC samples [38]. Importantly, a statistically significant correlation between Notch2 and Sox9 expression in all liver samples, as well as in ICC tumors was found (Sup Figs. 5A and 5B). Furthermore, analysis of the cBioPortal for Cancer Genomics dataset [39] revealed Notch2 amplification in 4/36 (11%) human ICC specimens, whereas no alterations were identified for Notch1 (Sup Fig. 5C). In humans, loss-of-function mutations in Notch2 or its ligand Jagged1 are associated with Alagille Syndrome, a developmental disorder leading to bile duct scarcity [40, 41]. These data support the prominent role of Notch2 in BEC differentiation and bile duct formation during development, and are consistent with the hypothesis that Notch2 may also control the BEC fate along human cholangiocarcinogenesis. The mechanisms whereby Notch2 promotes BEC differentiation and ICC development remain to be better delineated. Interestingly, the importance of the JNK signaling in biliary carcinogenesis has been recently demonstrated [30]. Of note, we found that the JNK pathway is impaired in Notch2-depleted livers (Fig. 5f), thus suggesting a functional crosstalk between these two cascades during ICC formation and/or progression. Additional investigation is necessary to unravel the function of the JNK signaling downstream of Notch2 in cholangiocarcinogenesis.

Unlike Notch2, the functional role of Notch1 in intrahepatic cholangiocarcinogenesis remains poorly understood. Using conditional *Notch1* KO mice, we showed that loss of Notch1 in tumor cells delays AKT/Yap-induced tumor development and mildly reduces the levels of a subset of Notch target genes. These results suggest that the contribution of Notch1 receptor to hepatocyte-derived ICC formation in mice might be limited. Intriguingly, IHC revealed that Notch1 is predominantly expressed in the tumor microenvironment (Figs. 2c and 4e). This staining pattern in AKT/Yap lesions is consistent with that in AKT/Ras liver tumors [24]. However, due to the low sensitivity of the anti-Notch1 antibody, we were unable to convincingly conclude whether Notch1 is expressed in ECs or tumor-associated fibroblast cells. Further studies, using single cell RNA-Seq analysis for instance, will be required to identify the cell types expressing Notch1. Furthermore, using proper cell-lineage Cre mouse lines in combination with conditional *Notch1* KO mice, it would be possible to unravel the precise mechanisms whereby Notch1 plays a role in cholangiocarcinogenesis. For instance, Lrat-Cre mice express Cre in fibroblasts, including hepatic stellate cells [42], and they can be used to delete *Notch1* in ICC-associated fibroblasts. Also, Tie2-Cre mice [43] could be used to delete *Notch1* in ECs. In summary, our investigation suggests that Notch1 acts in a paracrine fashion to regulate ICC development.

Finally, our study has important clinical implications. Targeting Notch has been proven highly toxic in tumor patients mainly at the gastrointestinal level to date, presumably due to the important physiological roles of various Notch-related proteins [14]. The present findings suggest that Notch2 might be a potential target in human ICC. Of note, promising anti-neoplastic effects by Notch2-specific antibodies were detected in ICC lesions from AKT/Ras mice [24]. Thus, further studies should be conducted to establish the eventual relevance of anti-Notch2 strategies for the treatment of this deadly disease.

## Materials and methods

### Constructs and reagents

We used constructs as described previously, including pT3-EF1α, pT3-EF1α-HA-myr-AKT (mouse), pT3-EF1α-YapS127A (human), pT3-EF1α-dnRBPJ (human), pCMV, pCMV-Cre, and pCMV/SB transposase [28, 44–46]. Adeno-associated virus encoding Cre-recombinase under the control of hepatocyte-specific thyroxine-binding globulin (Tbg) promoter (AAV8-Tbg-Cre) was provided by the University of Pennsylvania Vector Core (Philadelphia, PA). All plasmids were purified utilizing the Endotoxin Free Maxi prep kit (Sigma- Aldrich, St. Louis, MO).

### Animals

We obtained R26R-EYFP mice from Jackson Laboratory (Sacramento, CA, USA). Five female R26R-EYFP mice were injected with AAV8-Tbg-Cre. HA tagged AKT, YapS127 plasmid (AKT/Yap), and SB were delivered to the mice by hydrodynamic injection. FVB/N and *Notch2*^*flox/flox*^ mice were purchased from Jackson Laboratory. *Notch1*^*flox/flox*^ mice were a generous gift by Dr. Rong Wang at UCSF. Five female FVB/N mice were injected with AKT/Yap hydrodynamically; 10 male mice were randomly assigned to AKT/Yap/pT3 and AKT/Yap/dnRBPJ groups. A total of 9 female and 9 male *Notch2*^*flox/flox*^ mice were randomly assigned to AKT/Yap/pCMV and AKT/Yap/Cre groups. Finally, 7 female and 13 male *Notch1*^*flox/flox*^ mice were randomly assigned to AKT/Yap/pCMV and AKT/Yap/Cre cohorts. The study was not blinded.

### Hydrodynamic tail vein injection

We employed hydrodynamic injection as previously described in detail [47]. The dosage of pT3-EF1α-HA-myr-AKT (AKT) and pT3-EF1α-YapS127A (Yap) plasmids was 20 and 30 μg, respectively. To suppress the canonical Notch signaling, we applied 60 μg pT3-EF1α-dnRBPJ or 60 μg pT3-EF1α together with AKT and Yap plasmids in FVB/N mice. In transgenic models, we injected 60 μg pCMV-Cre or 60 μg empty pCMV vector together with AKT/Yap. Mice were housed, fed, and monitored according to protocols approved by the Committee for Animal Research at the University of California San Francisco (San Francisco, CA).

### Histology, IHC and IF

Samples were fixed overnight with Zinc Formal-Fixx (Thermo Shandon Limited, Runcorn) at 4 °C for subsequent paraffin-embedding. Sections were done at 5 μm in thickness. For IHC of HNF-4α, antigen unmasking was performed in Tris/EDTA buffer (pH 9.0), while for all other targets in sodium citrate buffer (pH 6.0). Other information could be found in the Supplementary Material and Methods.

### Protein extraction and western blot analysis

Specific protein extraction procedures can be found in Supplementary Material and Methods. Aliquots of 30 μg lysate were denatured by boiling in Tris–Glycine SDS Sample Buffer (Invitrogen, Grand Island, NY). We used SDS-PAGE for protein separation. And then protein was transferred onto nitrocellulose membranes (Invitrogen). Membranes were blocked in 5% non-fat dry milk in Tris-buffered saline containing 0.1% Tween 20 for 1 h and incubated with specific primary antibodies (Supplementary Table 1). Subsequently, a horseradish peroxidase-secondary antibody diluted 1:10,000 for 1 h was applied and revealed using the Super Signal West Pico Chemiluminescent Substrate (Pierce Chemical Co., New York, NY).

### RNA extraction and qPCR

We extracted total mRNA from liver tissues and cells using the Quick RNA miniprep kit (Zymo Research, Irvine, CA, USA). Next, mRNA expression was detected by qRT-PCR using SYBR Green Master Mix (Applied Biosystems, Foster City, CA, USA) in an QuantStudio™ 6 Flex system (Applied Biosystems). Expression of each gene was normalized with the 18S rRNA. Other conditions can be found in Supplementary Material and Methods. The list of primers is reported in Supplementary Table 2.

### Cell culture studies

We obtained the KKU-M213 and RBE ICC cell lines from the Japanese Collection of Research Bioresources Cell Bank (JCRB, Japan) and RIKEN cell bank (Tsukuba, Japan), respectively. HuCC-T1 was a generous gift by Dr. Gregory J. Gores (Mayo Clinic, Rochester, MN). HLE, SNU-449, and SNU-475 HCC cell lines were purchased from American Type Culture Collection (ATCC, Manassas, VA, USA). All cells were authenticated and tested clear of mycoplasma contamination. Cells were cultured separately in DMEM medium (Gibco, Grand Island, NY, USA) with 5% fetal bovine serum (Gibco), 100 μg/ml streptomycin, and 100 U/ml penicillin at 37 °C in 5% CO_2_ humidified incubator.

### In vitro siRNA transfection

KKU-M213, HuCC-T1, RBE ICC cell lines, and HLE, SUN-449, SNU-475 HCC cells were transfected with short interfering RNAs (siRNAs) targeting Notch2 (Thermo Fisher Scientific) or negative control siRNA (Thermo Fisher Scientific) using Lipofectamine RNA/iMAX (Thermo Fisher Scientific). Cells were harvested after 48 h for qPCR and Western blot analysis.

### Statistical analysis

The Prism 7.0 software (GraphPad, San Diego, CA) was used to analyze the data, which are presented as Means ± SD. Comparisons between two groups were performed with two-tailed unpaired *t* test. Welch correction was applied when necessary. *P* values < 0.05 were considered statistically significant.

## Electronic supplementary material

Supplementary Figures and Tables(PDF 937 kb)

Supplementary Material and Methods(DOCX 33 kb)
