# Supplementary material for: Notch2 controls hepatocyte-derived cholangiocarcinoma formation in mice
Source: Oncogene. 2018 Mar 16;37(24):3229–42. doi: 10.1038/s41388-018-0188-1 (PMC6002343; doi:10.1038/s41388-018-0188-1)
Supplement: Supplementary file 1 — Supplementary Figures and Tables(PDF 937 kb) [file 41388_2018_188_MOESM1_ESM.pdf]

Supplementary Figure 1

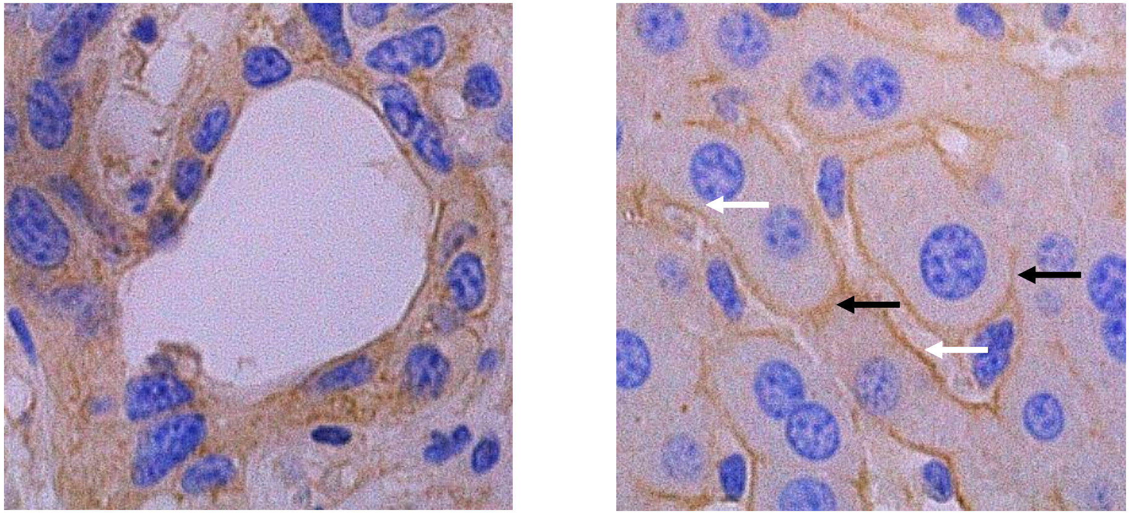

Sup. Figure 1: Notch1 Immunohistochemistry (IHC) of AKT/YapS127A tumors (**A**) or surrounding normal hepatocytes (**B**) with relatively long DAB incubation time. Weak cytoplasmic and membranous staining could be observed in ICC cells and normal hepatocytes. Black arrows point to hepatocyte membranous staining (with cell-cell connection). White arrows point to endothelial cell staining revealing sinusoids. Original magnification: 400x

Supplementary Figure 2

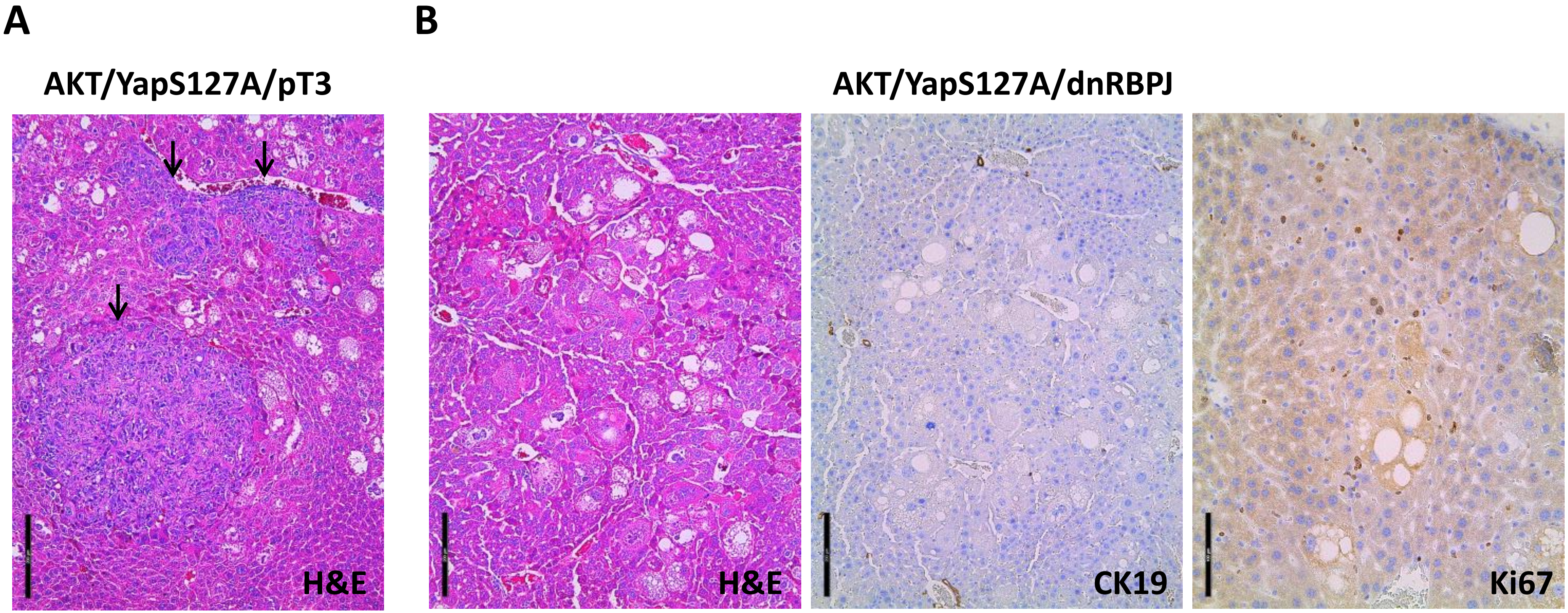

Sup. Figure 2: (A) Histology of AKT/Yap/pT3 and (**B)** AKT/Yap/dnRBPJ mice in the early stage of liver carcinogenesis, as assessed by hematoxylin and eosin (H&E) staining. Black arrows point to ICC formation in AKT/Yap/pT3 4.7wk mice, whereas AKT/Yap/dnRBPJ 4.7wk mice exhibit only mild steatosis. (**B)** Immunohistochemistry (IHC) of liver sections in AKT/Yap/dnRBPJ 4.7wk mice for CK19 and Ki67. No ICC lesions could be detected.

Supplementary Figure 3

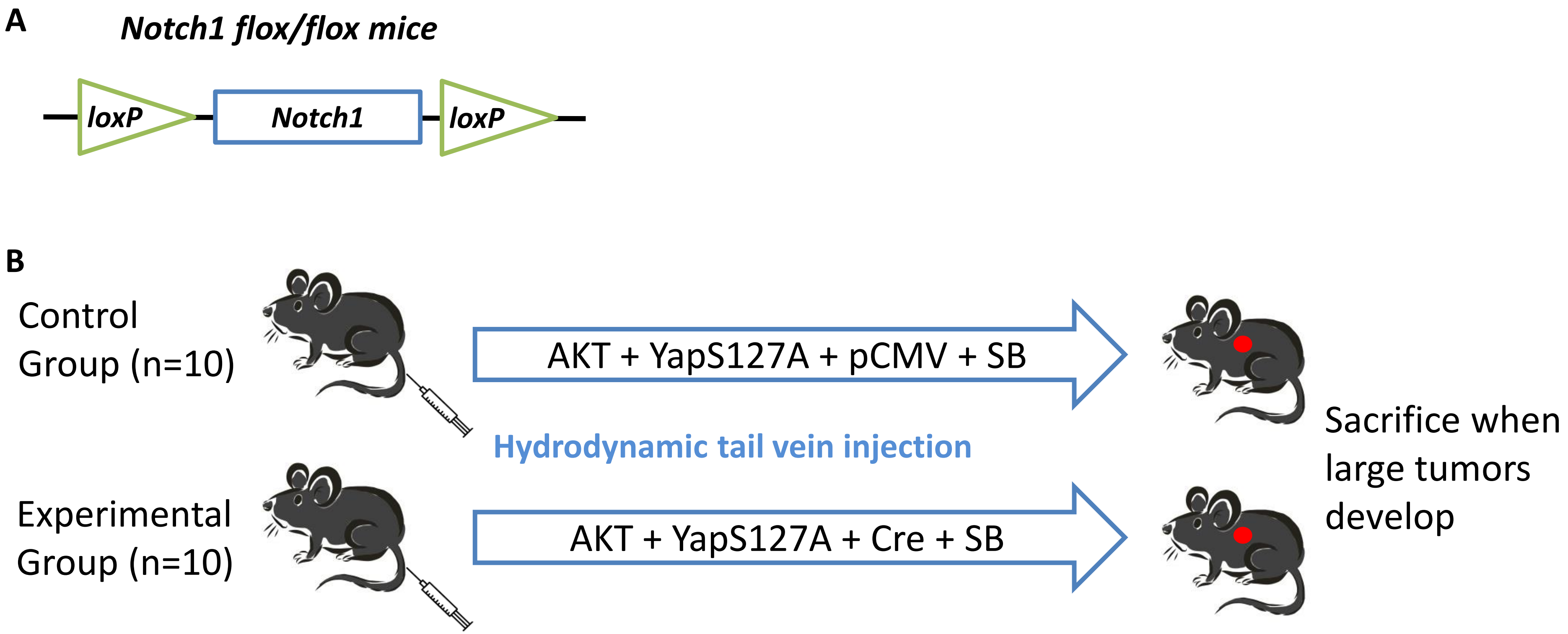

Sup. Figure 3: (A) The loxP site of *Notch1 flox/flox* mice. **(B)** Study design.

Supplementary Figure 4

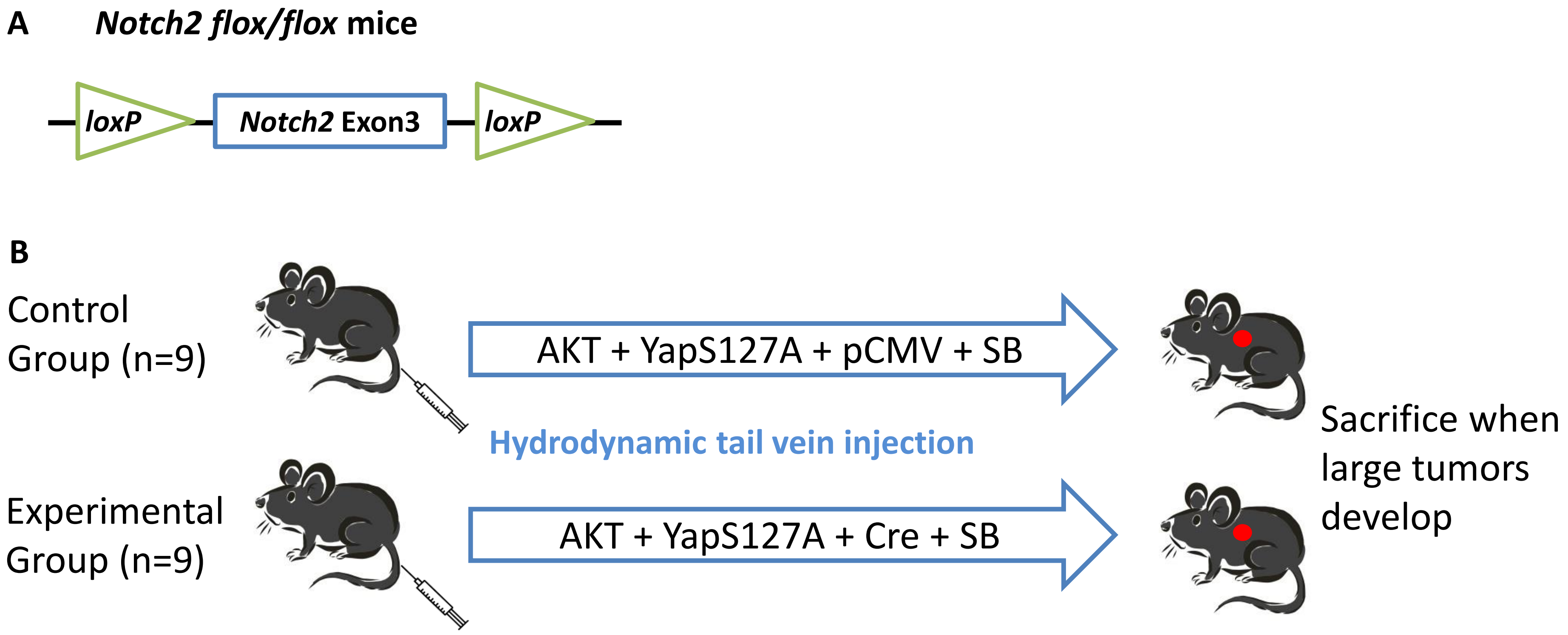

Sup. Figure 4: (A) The loxP site of *Notch2 flox/flox* mice. **(B)** Study design.

Supplementary Figure 5

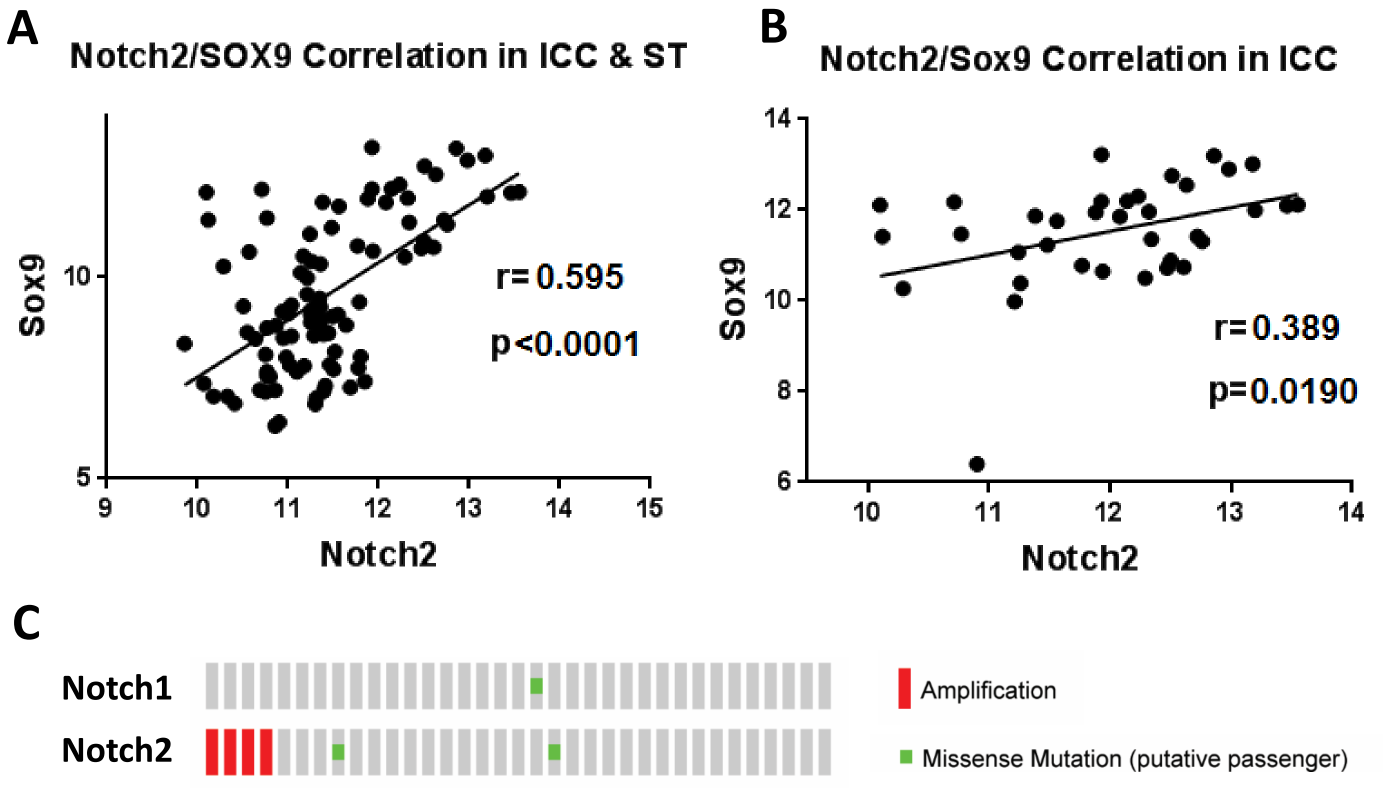

Sup. Figure 5: (A) Correlation analysis of Notch2 and SOX9 in human cholangiocarcinoma (ICC) and surrounding tissues (ST). **(B)** Correlation analysis of Notch2 and SOX9 only in human ICC. The data were analyzed by applying the Pearson correlation method. R and p value are presented on the graph. **(C)** Tumor samples with sequencing and CNA data from cBioPortal for Cancer Genomics showed amplification and missense mutation in Notch1 and Notch2 of 36 ICC patients.

Supplementary Table 1. Primary Antibodies for IHC, IF and WB

IHC	Concentration	Company	Catalogue No.
CK19	1:500	Abcam	Ab133496
HNF-4α	1:2000	Abcam	Ab181604
CD34	1:2500	Abcam	Ab81289
Ki67	1:150	Thermo Fisher scientific	RM-9106-S1
Notch1	1:200	Proteintech	20678-I-AP
Notch2	1:80	Cell Signaling Technology	5732
Vimentin	1:100	Cell Signaling Technology	5741
**IF**	**Concentration**	**Company**	
CK19	1:500	Abcam	Ab52625
GFP	1:2000	Abcam	Ab13970
HA-tag	1:100	Cell Signaling Technology	2367
Alexa Fluor	1:500	Invitrogen	A11040; A11008;
**WB**	**Concentration**	**Company**	
Notch1	1:1000	Cell Signaling Technology	3608
Notch2	1:1000	Cell Signaling Technology	5732
Jagged1	1:2000	Abcam	Ab109536
Yap	1:1000	Cell Signaling Technology	4912
SOX9	1:5000	Abcam	Ab185230
Total AKT	1:2000	Cell Signaling Technology	9272
p-AKT ^{S473}	1:1000	Cell Signaling Technology	3787
HA-tag	1:1000	Cell Signaling Technology	2367
p-JNK	1:1000	Cell Signaling Technology	4668
JNK	1:1000	Cell Signaling Technology	9252
GAPDH	1:10000	EMD Millipore	AB2302

Supplementary Table 2. Primers used in qRT-PCR

	Forward	Reverse
18s rRNA	CGGCTACCACATCCAAGGAA	GCTGGAATTACCGCGGCT
Mouse-Notch1	CCCTTGCTCTGCCTAACGC	GGAGTCCTGGCATCGTTGG
Mouse-Notch2	ATGTGGACGAGTGTCTGTTGC	GGAAGCATAGGCACAGTCATC
Mouse-Jagged1	CCTCGGGTCAGTTTGAGCTG	CCTTGAGGCACACTTTGAAGTA
Mouse-Jagged2	CAATGACACCACTCCAGATGAG	GGCCAAAGAAGTCGTTGCG
Mouse-Hes1	AAAGCCTATCATGGAGAAGAGGCG	GGAATGCCGGGAGCTATCTTTCTT
Mouse-Hes5	AAGCGCCCTTGCGAGGAAAC	GGTAGTTGTCGGTGAATTGGAC
Mouse-Hey1	GCGCGGACGAGAATGGAAA	TCAGGTGATCCACAGTCATCTG
Mouse-Hey2	AAGCGCCCTTGTGAGGAAAC	GGTAGTTGTCGGTGAATTGGAC
Mouse-HeyL	CAGCCCTTCGCAGATGCAA	CCAATCGTCGCAATTCAGAAAG
Mouse-AFP	TCTGCTGGCACGCAAGAAG	TCGGCAGGTTCTGGAAACTG
Mouse-GPC3	CAGCCCGGACTCAAATGGG	CAGCCGTGCTGTTAGTTGGTA
Human-Notch2	GATCACCCGAATGGCTATGAAT	GGGGTCACAGTTGTCAATGTT
Human-Nrarp	TCAACGTGAACTCGTTCGGG	ACTTCGCCTTGGTGATGAGAT
Human-Epcam	GCGGCTCAGAGAGACTGTG	CCAAGCATTTAGACGCCAGTTT
Human-Hes1	GTGAAGCACCTCCGGAAC	CGTTCATGCACTCGCTGA
Human-Hey1	GTTCGGCTCTAGGTTCCATGT	CGTCGGCGCTTCTCAATTATTC
