# Supplementary material for: Notch2 controls hepatocyte-derived cholangiocarcinoma formation in mice
Source: Oncogene. 2018 Mar 16;37(24):3229–42. doi: 10.1038/s41388-018-0188-1 (PMC6002343; doi:10.1038/s41388-018-0188-1)
Supplement: Supplementary file 2 — Supplementary Material and Methods(DOCX 33 kb) [file 41388_2018_188_MOESM2_ESM.docx]

#### Supplementary Material and Methods Constructs and Reagents

The constructs used for injection, including pT3-EF1α, pT3-EF1α-HA-myr-AKT (mouse), pT3- EF1α-YapS127A (human), pT3-EF1α-dnRBPJ (human), pCMV, pCMV-Cre and pCMV/sleeping beauty transposase (SB), were described previously [1,](#bookmark0) [2,](#bookmark1) [3,](#bookmark2) [4.](#bookmark3) Adeno-associated virus encoding Cre-recombinase under the control of hepatocyte specific thyroxine-binding globulin (Tbg) promoter (AAV8-Tbg-Cre) was obtained from University of Pennsylvania Vector Core (Philadelphia, PA). Plasmids were purified using the Endotoxin Free Maxi prep kit (Sigma- Aldrich, St. Louis, MO) before being injected into the mice.

#### Animals

At least five mice per group were used in the experiments when they reached the age of six weeks. R26R-EYFP mice were obtained from Jackson Laboratory (Sacramento, CA, USA). A total of 5 female R26R-EYFP mice were injected with AAV8-Tbg-Cre. Then hydrodynamic tail vein injection was delivered with hemagglutinin (HA) tagged AKT, YapS127 plasmid (AKT/Yap) and Sleeping Beauty (SB). FVB/N and *Notch2flox/flox* mice were also obtained from Jackson Laboratory. A total of 5 female FVB mice were injected with AKT/Yap hydrodynamically. And a total of 10 male mice were randomly assigned to AKT/Yap/pT3 and AKT/Yap/dnRBPJ group. A

total of 9 female and 9 male *Notch2flox/flox* mice were randomly assigned into AKT/Yap/pCMV and AKT/Yap/Cre group. *Notch1flox/flox* mice were generously provided from Dr. Rong Wang at UCSF. A total of 7 female and 13 male *Notch1flox/flox* mice were randomly assigned into AKT/Yap/pCMV and AKT/Yap/Cre group. The study was not blinded.

#### Cell culture

ICC cell line KKU-M213 was originally obtained from Japanese Collection of Research Bioresources Cell Bank (JCRB, Japan) and RBE from RIKEN cell bank (Tsukuba, Japan).

HuCC-T1 was generously gifted by Dr. Gregory J. Gores from Mayo Clinic. HCC cell lines HLE, SUN-449, SNU-475 were obtained from American Type Culture Collection (ATCC, Manassas, VA, USA). All cells were authenticated and tested clear of mycoplasma contamination. All cells were cultured separately in DMEM medium (Gibco, Grand Island, NY, USA) with 5% fetal bovine serum (Gibco), 100 μg/ml streptomycin and 100 U/ml penicillin at 37°C in 5% CO2 humidified incubator.

#### Histology and Immunohistochemistry (IHC) Analysis

Samples for immunostainings were fixed overnight with Zinc Formal-Fixx (Thermo Shandon Limited, Runcorn) at 4°C for subsequent paraffin-embedding. Sections were done at 5 μm in thickness. For IHC of HNF-4α, antigen retrieval was performed in Tris/EDTA buffer (pH 9.0). For IHC of all other targets, antigen retrieval was performed in sodium citrate buffer (pH 6.0) by placement in a microwave on high for 10 min, followed by a 20-min cool down at room temperature. After a blocking step with the 5% goat serum and Avidin-Biotin blocking kit (Vector Laboratories, Burlingame, CA), the slides were incubated with primary antibodies (Supplementary Table 1) overnight at 4°C. Slides were then subjected to 3% hydrogen peroxide for 10 min to quench endogenous peroxidase activity. Subsequently, the biotin conjugated secondary antibody (Life Technology, Waltham, MA) was applied at a 1:500 dilution for 30 min at room temperature. Immunostainings were visualized using the Vectastain Elite ABC Kit (Vector Laboratories) and DAB substrates (Dako North America, Carpinteria, CA). Slides were counterstained with hematoxylin. CK19 and Ki67 staining was quantified using ImageJ (NIH) and Image Pro Plus (Media Cybernetics, Rockville, MD), respectively.

#### Immunofluorescent (IF) Analysis

Sections were also done at 5 μm in thickness. Antigen retrieval was performed in sodium citrate buffer (pH 6.0) by placement in a microwave on high for 10 min, followed by a 20-min cool down

at room temperature. After blocking in 10% serum for 1 hour, sections were incubated with primary antibodies overnight at 4°C and secondary antibodies for 1 hour at room temperature (Supplemental Tables 1). Nuclear DNA was stained with 300 nM DAPI (Millipore).

#### Protein Extraction and Western Blot Analysis

Frozen mouse liver specimens were homogenized, and cultured cell samples were lysed in Mammalian Protein extraction reagent containing the Complete Protease Inhibitor Cocktail (Thermo Scientific, Waltham, MA, USA). The lysate was clarified by centrifugation (14,000 rpm for 10 minutes at 4°C), and stored at -80°C until assayed. Protein concentrations were determined with the Bio-Rad Protein Assay Kit (Bio-Rad, Hercules, CA) using bovine serum albumin as standard. Aliquots of 30μg lysate were denatured by boiling in Tris-Glycine SDS Sample Buffer (Invitrogen), separated by SDS-PAGE, and then transferred onto nitrocellulose membranes (Invitrogen, Grand Island, NY). Membranes were blocked in 5% non-fat dry milk in Tris-buffered saline containing 0.1% Tween 20 for 1 hour and probed with specific antibodies listed in Supplementary Table 1. Each primary antibody was followed by incubation with horseradish peroxidase-secondary antibody diluted 1:10,000 for 1 hour and then revealed with the Super Signal West Pico Chemiluminescent Substrate (Pierce Chemical Co., New York, NY).

#### RNA extraction and qPCR

Total mRNA was extracted from liver tissues and cells using Quick RNA miniprep kit (Zymo Research, Irvine, CA, USA). mRNA expression was determined by qRT-PCR using SYBR Green Master Mix (Applied Biosystems, Foster City, CA, USA) in an QuantStudio™ 6 Flex system (Applied Biosystems). Expression of each specific gene mRNA by cells was normalized with the 18S rRNA. Thermal cycling conditions included an initial hold period at 95°C for 10 minutes, which was followed by a three-step PCR program of 95°C for 15 seconds, 60°C for 1 min and 72°C for 30 seconds for a total of 40 cycles. Primers used in this study are shown in

Supplementary Table 2.

**Reference**

1. Tao J, Calvisi DF, Ranganathan S, Cigliano A, Zhou L, Singh S*, et al.* Activation of beta-catenin and Yap1 in human hepatoblastoma and induction of hepatocarcinogenesis in mice. *Gastroenterology* 2014, **147**(3)**:** 690-701.
2. Marti P, Stein C, Blumer T, Abraham Y, Dill MT, Pikiolek M*, et al.* YAP promotes proliferation, chemoresistance, and angiogenesis in human cholangiocarcinoma through TEAD transcription factors. *Hepatology* 2015, **62**(5)**:** 1497-1510.
3. Hu J, Che L, Li L, Pilo MG, Cigliano A, Ribback S*, et al.* Co-activation of AKT and c-Met triggers rapid hepatocellular carcinoma development via the mTORC1/FASN pathway in mice. *Scientific reports* 2016, **6:** 20484.
4. Che L, Fan B, Pilo MG, Xu Z, Liu Y, Cigliano A*, et al.* Jagged 1 is a major Notch ligand along cholangiocarcinoma development in mice and humans. *Oncogenesis* 2016, **5**(12)**:** e274.
